# Supplementary material for: Histones and histone variant families in prokaryotes
Source: Nat Commun. 2024 Sep 11;15:7950. doi: 10.1038/s41467-024-52337-y (PMC11390915; doi:10.1038/s41467-024-52337-y)
Supplement: Supplementary file 3 — Reporting Summary [file 41467_2024_52337_MOESM3_ESM.pdf]

nature portfolio

Corresponding author(s): Samuel Schwab, Yimin Hu, Bert van Erp, Marc K. M. Cajili, Marcus D. Hartmann, Birte Hernandez Alvarez, Vikram Alva, Aimee L. Boyle, Remus T. Dame

Last updated by author(s): Aug 8, 2024

Reporting Summary

Nature Portfolio wishes to improve the reproducibility of the work that we publish. This form provides structure for consistency and transparency in reporting. For further information on Nature Portfolio policies, see our Editorial Policies and the Editorial Policy Checklist.

Statistics

For all statistical analyses, confirm that the following items are present in the figure legend, table legend, main text, or Methods section.

n/a	Confirmed	
☐	☒	The exact sample size (*n*) for each experimental group/condition, given as a discrete number and unit of measurement
☐	☒	A statement on whether measurements were taken from distinct samples or whether the same sample was measured repeatedly
☒	☐	The statistical test(s) used AND whether they are one- or two-sided<br>*Only common tests should be described solely by name; describe more complex techniques in the Methods section.*
☒	☐	A description of all covariates tested
☒	☐	A description of any assumptions or corrections, such as tests of normality and adjustment for multiple comparisons
☐	☒	A full description of the statistical parameters including central tendency (e.g. means) or other basic estimates (e.g. regression coefficient) AND variation (e.g. standard deviation) or associated estimates of uncertainty (e.g. confidence intervals)
☒	☐	For null hypothesis testing, the test statistic (e.g. *F*, *t*, *r*) with confidence intervals, effect sizes, degrees of freedom and *P* value noted<br>*Give P values as exact values whenever suitable.*
☒	☐	For Bayesian analysis, information on the choice of priors and Markov chain Monte Carlo settings
☒	☐	For hierarchical and complex designs, identification of the appropriate level for tests and full reporting of outcomes
☒	☐	Estimates of effect sizes (e.g. Cohen's *d*, Pearson's *r*), indicating how they were calculated

Our web collection on statistics for biologists contains articles on many of the points above.

Software and code

Policy information about availability of computer code

Data collection	To predict the monomer and multimer structures, AlphaFold2, AlphaFold2-multimer (v2), (Local)Colabfold (v1.3) were used.
Data analysis	Phylogenetic trees were generated with RAxML Next Generation (v1.1.0). The trees were visualised in R (v4.1.0) with ggtree (v3.2.1).<br>HMM profiles were generated with HMMER (v3.3.2). Alignments for the HMM profiles were made with Muscle (v5). Logos of the HMM profiles were made with Skylign (http://skylign.org/).<br>Clustering of histone sequences was done in CLANS (https://toolkit.tuebingen.mpg.de/tools/clans).<br>Structural similarity searches against UniProt and the PDB were performed on the webserver of FoldSeek (https://search.foldseek.com/search).<br>Gene clustering was performed with Clinker (v0.0.26).<br>Crystal structure were solved with MOLREP and refined in COOT and REFMAC5.<br>Prediction of transmembrane probability and topology were performed with DeepTMHMM.<br>Protein structures were analysed and visualised with ChimeraX (v1.6)<br>Custom code is available on Github: https://github.com/SamuelSchwab/Histones-and-histone-variant-families-in-prokaryotes

For manuscripts utilizing custom algorithms or software that are central to the research but not yet described in published literature, software must be made available to editors and reviewers. We strongly encourage code deposition in a community repository (e.g. GitHub). See the Nature Portfolio guidelines for submitting code & software for further information.

Data

Policy information about availability of data

All manuscripts must include a data availability statement. This statement should provide the following information, where applicable:

- Accession codes, unique identifiers, or web links for publicly available datasets
- A description of any restrictions on data availability
- For clinical datasets or third party data, please ensure that the statement adheres to our policy

The list of prokaryotic histones, the predicted AlphaFold structures shown in this article, the HMM profiles, and the DNA-bridging data are available from the 4TU repository (https://data.4tu.nl) at doi.org/10.4121/d268a6a9-2fc5-46aa-a236-0ca3d3f7ed75. X-ray structural data was deposited in the PDB database with accession number 9F2C [https://www.rcsb.org/structure/9f2c].

Research involving human participants, their data, or biological material

Policy information about studies with human participants or human data. See also policy information about sex, gender (identity/presentation), and sexual orientation and race, ethnicity and racism.

Reporting on sex and gender	No human participants were involved; no human data was used.
Reporting on race, ethnicity, or other socially relevant groupings	No human participants were involved; no human data was used.
Population characteristics	No human participants were involved; no human data was used.
Recruitment	No human participants were involved; no human data was used.
Ethics oversight	No human participants were involved; no human data was used.

Note that full information on the approval of the study protocol must also be provided in the manuscript.

Field-specific reporting

Please select the one below that is the best fit for your research. If you are not sure, read the appropriate sections before making your selection.

☒ Life sciences ☐ Behavioural & social sciences ☐ Ecological, evolutionary & environmental sciences

For a reference copy of the document with all sections, see nature.com/documents/nr-reporting-summary-flat.pdf

Life sciences study design

All studies must disclose on these points even when the disclosure is negative.

Sample size	Three independent measurements with seperate samples were performed for the DNA-bridging assay.
Data exclusions	No data was excluded.
Replication	The measurement was repeated three times with independent samples. To see the standard deviation of these three measurements, see Suppllementary Figure 25b.
Randomization	Randomization is not relevant for this study.
Blinding	Blinding is not relevant for this study.

Reporting for specific materials, systems and methods

We require information from authors about some types of materials, experimental systems and methods used in many studies. Here, indicate whether each material, system or method listed is relevant to your study. If you are not sure if a list item applies to your research, read the appropriate section before selecting a response.

Materials & experimental systems

n/a	Involved in the study
☒	☐ Antibodies
☒	☐ Eukaryotic cell lines
☒	☐ Palaeontology and archaeology
☒	☐ Animals and other organisms
☒	☐ Clinical data
☒	☐ Dual use research of concern
☒	☐ Plants

Methods

n/a	Involved in the study
☒	☐ ChIP-seq
☒	☐ Flow cytometry
☒	☐ MRI-based neuroimaging

Plants

Seed stocks	No plants were used.
Novel plant genotypes	No plants were used.
Authentication	No plants were used.
